# Artificial lipidation of proteins and peptides: from mechanism to clinical applications

**DOI:** 10.1111/febs.70298

**Published:** 2025-10-23

**Authors:** Jiaming Mu, Emily Vong, Sheiliza Carmali

**Affiliations:** ^1^ School of Pharmacy Queen's University Belfast UK

**Keywords:** albumin binding, drug delivery, half‐life extension, protein lipidation, therapeutic peptides

## Abstract

The landscape of modern medicine has been transformed by protein‐based therapeutics, offering targeted treatments for complex disorders with remarkable specificity and efficacy. However, these biologics face significant limitations in clinical settings, including rapid clearance, vulnerability to enzymatic degradation, poor absorption across biological membranes and inefficient distribution within target tissues. Artificial lipidation provides an innovative solution to these challenges, by the deliberate attachment of lipid groups to proteins and peptide structures. This biomimetic approach harnesses principles observed in natural post‐translational modifications to create therapeutics with superior pharmacological profiles. By strategically incorporating lipid moieties, researchers can significantly prolong circulation half‐life through albumin binding, protect against proteolytic breakdown, facilitate cellular uptake, customize pharmacokinetic parameters and enhance tissue‐specific targeting. This Review provides a comprehensive analysis of current lipidation technologies, contrasting covalent modification strategies with noncovalent complexation approaches. We examine the molecular mechanisms underlying the therapeutic benefits, survey successful clinical applications and explore emerging opportunities across diverse therapeutic areas. Through this analysis, we offer insights to guide rational design decisions for developing optimized lipidated biotherapeutics with enhanced clinical performance.

## Graphical abstract

Artificial lipidation transforms protein and peptide therapeutics by attaching fatty acid chains through reversible or permanent chemical strategies. This biomimetic approach delivers four key therapeutic advantages: extended circulation times (enabling weekly instead of daily dosing), alternative administration routes including oral delivery, precise tissue targeting and enhanced protection against degradation. These benefits have revolutionized diabetes treatment and are expanding across diverse therapeutic areas.

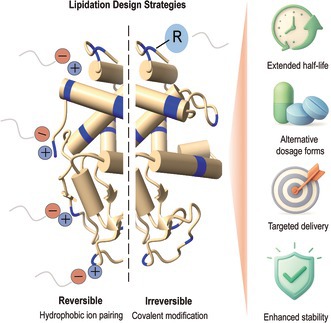

**Abbreviations**
ADO: 8‐amino‐3,6‐dioxaoctanoic acid
BBB: blood–brain barrier
DPP‐4: dipeptidyl peptidase‐4
EU: European Union
FcRn: neonatal fragment crystallizable receptors
FDA: Food and Drug Administration
GHD: growth hormone deficiency
GLP‐1: glucagon‐like peptide 1
hGH: human growth hormone
HIP: hydrophobic ion pairing
HIV‐1: human immunodeficiency virus type 1
HSA: human serum albumin
MHC: major histocompatibility complex
MTG: microbial transglutaminase
NHS: *N*‐hydroxysuccinimide
NMT: *N*‐myristoyltransferase
pI: isoelectric point
PLGA: poly lactide‐co‐glycolic acid

## Introduction

Protein therapeutics have revolutionized modern medicine by enabling precise treatments for complex diseases with unprecedented specificity and potency [1, 2]. These biologics now represent over 35% of newly approved drugs, with global market revenues exceeding $300 billion annually [3, 4]. Despite their commercial success, clinical applications remain constrained by fundamental challenges, including rapid systemic clearance, susceptibility to proteolytic degradation, limited membrane permeability and poor tissue distribution [5, 6].

Drawing inspiration from Nature's own molecular engineering, lipid modification has emerged as a transformative strategy to overcome these barriers, offering a versatile toolkit to enhance protein drug delivery and therapeutic efficacy [7, 8]. In biological systems, lipidation serves as an intricate molecular switch, precisely orchestrating protein function through post‐translational modifications [9, 10]. These modifications control diverse cellular processes by anchoring proteins to specific membrane domains, facilitating protein–protein interactions, modulating conformational dynamics and regulating cellular trafficking pathways [11, 12, 13].

Artificial lipidation represents an elegant bioengineering approach that strategically adapts and expands upon these natural principles [14]. Through rational design of lipid modifications, employing both covalent and noncovalent strategies, researchers can (a) extend plasma circulation times via enhanced protein–carrier interactions [15], (b) improve metabolic stability through altered protease accessibility [16], (c) enhance cellular membrane permeability through optimiszed amphipathicity [17], (d) fine‐tune pharmacokinetic properties through systemic lipid variation and (e) enable targeted drug delivery via specific tissue interactions [18].

**Fig. 1:**
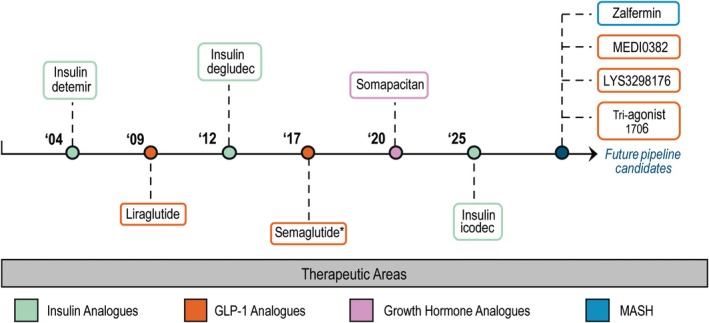
Clinical timeline for approved and currently in development lipidated therapeutics from 2004 to 2025, categorized by therapeutic area. Insulin analogues are shown in green, GLP‐1 analogues in orange, growth hormone analogues in pink and therapeutic targeting metabolic‐associated steatohepatitis in blue. Lipidation design progress has led to improved dosing regimens (e.g., Liraglutide (C16) requires daily dosing while Semaglutide (C18) is administered weekly). *Semaglutide was first approved by the FDA for type 2 diabetes in 2017 and for weight loss in 2021.

The clinical success of artificial lipidation is powerfully demonstrated through several breakthrough therapeutics (Fig. 1) [19]. The development of insulin detemir (Levemir^®^) established the foundation for next‐generation glucagon‐like peptide 1 (GLP‐1) receptor agonists with fatty acid chain length serving as a key design parameter for controlling circulation time and dosing frequency [20]. These advances demonstrate how precise control of lipidation, alone or in combination with other molecular modifications, can predictably engineer desired dosing regimens and enhance therapeutic outcomes.

However, the successful development of lipidated therapeutics faces several challenges. Manufacturing scale‐up, maintaining protein stability during conjugation and controlling the degree of modification require careful optimization. Additionally, regulatory requirements add another level of complexity, demanding rigorous characterization and safety assessments. Despite these challenges, the field continues to expand beyond diabetes and metabolic disorders into diverse therapeutic areas, including oncology, autoimmune conditions and rare genetic disorders.

This review examines the current state of artificial peptide and protein lipidation, beginning with lipidation strategies (Section Lipidation strategies: the chemical toolbox), followed by mechanisms of action and physicochemical effects (Section From structure to function: how lipidation transforms therapeutic properties), therapeutic and clinical implications (Section Therapeutic applications and implications) and concluding with challenges and outlook including emerging technologies and market opportunities (Section Challenges and future perspectives). While we provide comprehensive coverage of both covalent and noncovalent approaches, our focus remains on therapeutic applications rather than diagnostic or analytical uses of lipidated proteins. We analyse key success factors in clinical translation, discuss ongoing challenges and highlight emerging opportunities in this rapidly evolving field. Our insights aim to guide the development of next‐generation biotherapeutics through rationalization of lipid modifications.

## Lipidation strategies: the chemical toolbox

**Fig. 2:**
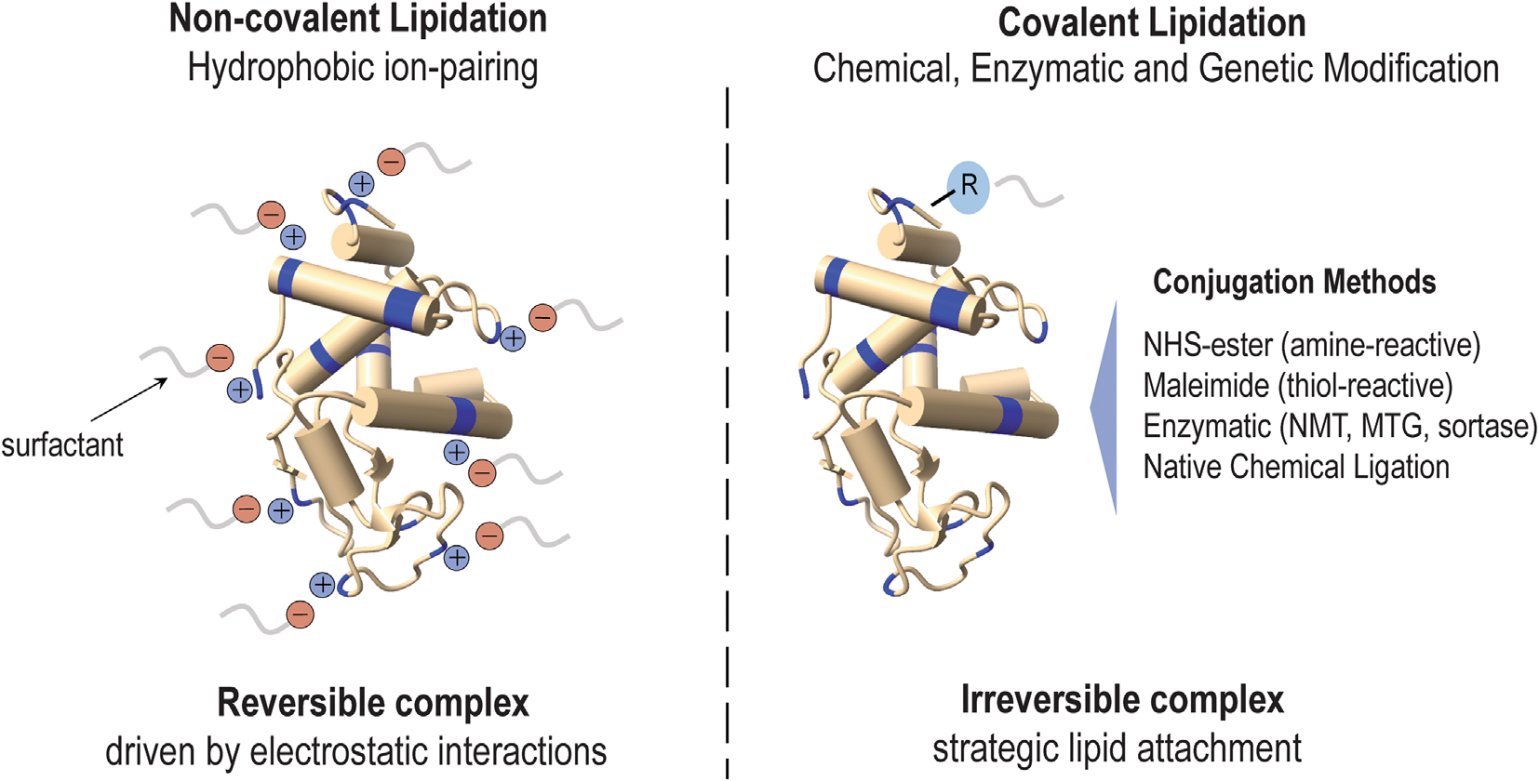
Molecular mechanisms of protein lipidation strategies. Left panel: Noncovalent lipidation through hydrophobic ion pairing, where surfactant molecules (shown with negative charges in red) interact electrostatically with positively charged regions (shown in blue) on the protein surface, forming reversible complexes. Right panel: Covalent lipidation, achieved by chemical, enzymatic or genetic modification, is where lipid moieties containing reactive groups are strategically and permanently attached to specific sites on the protein, creating irreversible complexes. MTG, microbial transglutaminase; NMT, *N*‐myristoyltransferase.

Lipidation, the addition of hydrocarbon chains of various lengths to proteins or peptides, can be achieved through two distinct strategies: noncovalent association and covalent modification (Fig. 2). The choice between these approaches is guided by rational design principles that consider protein and peptide structure, desired pharmacokinetic profile, administration route and manufacturing processes.

### Noncovalent lipidation: harnessing hydrophobic ion pairing

Hydrophobic ion pairing (HIP) is an elegant strategy that enhances molecular lipophilicity without chemical modification [21, 22]. It involves the stoichiometric association between charged molecules and oppositely charged surfactants containing hydrophobic domains. For proteins and peptides, this interaction primarily occurs through basic amino acid residues—lysine, arginine and histidine—which carry positive charges below their isoelectric point (pI) [22].

The formation of HIP is governed by several critical parameters including pH, ionic strength and surfactant properties [23]. The protein's net charge and isoelectric point determine suitable pH conditions for complexation, with most therapeutic proteins having pI values between 4.0 and 7.0 [24]. At pH values below the protein's pI, protonation of basic residues creates a net positive charge that facilitates interaction with anionic surfactants. Surface charge can be strategically adjusted by controlling pH relative to the protein's pI, with optimal conditions typically maintaining a pH difference of at least 2 units from both surfactant p*K*a and protein pI to ensure efficient ionization while maintaining protein stability [25].

Surfactant selection is equally critical for protein applications. Both surfactant p*K*a and partition coefficient (Log *P*) influence complex formation and stability. Sulphate and sulphonate‐based surfactants, such as sodium dodecyl sulphate and dioctyl sulfosuccinate, provide stable anionic counterions due to their low p*K*a values in comparison to carboxylate‐based alternatives [22]. The hydrophobic chain length and saturation directly influence complex lipophilicity, with Log *P* values greater than 2 typically required for stable incorporation into nanocarrier systems [23].

HIP has enabled researchers to encapsulate peptide drugs that were previously difficult to incorporate into lipophilic carriers or to substantially improve the encapsulation efficiency in existing systems. Key examples include the ion pairing of exenatide with n‐octadecyl sulphate before incorporation into a self‐emulsifying drug delivery system [26], complexation of insulin with sodium deoxycholate for poly(lactide‐co‐glycolic acid) (PLGA) nanoparticle delivery [27], and ion pairing of leuprolide acetate with sodium oleate for microsphere encapsulation [28]. The approach has demonstrated substantial improvements in loading efficiency and bioavailability across multiple delivery platforms.

The primary advantage of HIP is its ability to overcome delivery challenges for hydrophilic therapeutics by reversibly binding them with hydrophobic counterions, making them more amenable to encapsulation in lipophilic carriers [29]. Research has shown that decomplexation typically preserves bioactivity and in some cases even improves activity due to favourable surfactant‐induced conformational changes [25, 30]. From a regulatory standpoint, HIP might provide benefits compared to covalent modification strategies, as it can be viewed a formulation method rather than the development of a new molecular entity [31, 32].

However, several challenges must be considered when implementing HIP for protein delivery. The release kinetics require careful management, as decomplexation is driven by salt‐induced counterion competition and pH‐dependent charge negation [33, 34]. Additionally, some HIP complexes may assemble on the surface of lipid‐based nanocarriers rather than being fully encapsulated, potentially exposing them to peptidases and proteases due to their pronounced hydrophilic–lipophilic structure and compromising stability in biological environments [35].

### Covalent lipidation: strategic formation of stable bonds

Covalent lipidation enhances peptide and protein delivery through the strategic attachment of lipid moieties via stable chemical bonds [36]. This approach systematically addresses fundamental limitations of protein and peptide therapeutics by creating conjugates with substantially improved pharmacological properties, including increased stability against enzymatic degradation, extended circulation times, improved bioavailability and optimized biodistribution across target tissues.

#### Lipid selection and site targeting: critical design parameters

Lipid selection is a critical decision in designing lipidated therapeutics, as it significantly influences the pharmacological outcome of the modified protein or peptide. Different lipid structures enable specific enhancements in stability, cell adhesion or circulation half‐life, allowing tailored optimization of therapeutic properties.

Fatty acids are the most widely used class of lipid modifications, offering a spectrum of chain lengths that enable fine‐tuned control over protein properties [37, 38]. Short‐chain variants like caprylic acid (C8) provide enhanced water solubility while maintaining modest lipophilic character. Medium‐chain fatty acids, particularly myristic acid (C14), facilitate protein–membrane interactions through *N*‐myristoylation of N‐terminal glycine residues, playing crucial roles in protein localization and function. Palmitic acid (C16) represents one of the most prevalent fatty acid modifications, with S‐palmitoylation enabling reversible cysteine modification that influences protein trafficking and stability. Longer‐chain variants like stearic acid (C18) provide increased hydrophobicity and stronger serum albumin binding, typically resulting in extended circulation times.

The influence of fatty acid chain length on therapeutic performance is well documented. Research by Madsen *et al*. [20] demonstrated that longer fatty acid chains create stronger albumin interactions, systematically extending GLP‐1 half‐life from 0.8 h (C10) to 16 h (C16) and up to 21 h (C18). This relationship occurs because longer hydrophobic chains bind more tightly to albumin's fatty acid binding sites, creating a circulating reservoir that slowly releases active drug. Building on these foundational structure–activity studies, Knudsen and Lau [39] demonstrated that acylation of GLP‐1 with C16 (palmitoyl) optimally prolonged action while maintaining biological potency, leading to the development of liraglutide. Subsequent studies by Lau *et al*. [40] explored alternative fatty diacid modifications, culminating in the C18 diacid modification of semaglutide with extended half‐life properties. This approach has demonstrated notable success with salmon calcitonin, where palmitic acid attachment to specific cysteine residues significantly enhanced oral bioavailability [41].

Beyond fatty acids, several other lipid classes offer unique advantages. Prenylation, involving the irreversible attachment of isoprenoid units (ranging from 10 to 20 carbons), enhances membrane interactions through thioether bonds [42]. Cholesterol conjugation provides another strategy for enhancing membrane interactions and cellular uptake [43].

Site selection is a key determinant of therapeutic outcomes. Modification near a protein's active site can significantly impact biological function through steric effects, either enhancing or diminishing activity through conformational changes [44]. The modification site also influences protein stability, with properly positioned lipids facilitating membrane interactions and providing protection against proteolytic degradation. Strategic positioning can enhance serum albumin binding and circulation time while maintaining optimal solubility. This balance proves particularly important, as inappropriate site selection can promote unwanted aggregation or provoke adverse immune responses *in vivo* [19, 45].

#### Conjugation chemistry: methods for precision lipidation

Chemical approaches to protein lipidation offer precise control over modification through various reactive chemistries [14]. *N*‐hydroxysuccinimide (NHS) ester chemistry remains the most widely employed strategy for amine‐reactive modification, particularly targeting lysine residues. This approach has driven the development of several successful therapeutics, including insulin detemir's myristic acid attachment at LysB29, which enables albumin binding and prolonged circulation, and insulin icodec with a sophisticated C20 diacid modification to achieve once‐weekly dosing [46].

Alternative chemical approaches include maleimide chemistry for thiol‐selective conjugation, particularly useful for targeting unpaired cysteine residues [47]. Native chemical ligation represents another powerful technique for site‐specific lipidation, particularly for N‐terminal modifications [48].

Enzymatic lipidation offers advantages of high specificity and mild reaction conditions. N‐myristoyltransferase (NMT) catalyses the transfer of myristoyl groups to N‐terminal glycine residues with high fidelity, mimicking natural post‐translational modifications [49]. Sortase‐mediated conjugation uses the transpeptidase activity of sortase enzymes to catalyse the formation of a peptide bond between a C‐terminal recognition sequence (LPXTG) and an N‐terminal glycine, resulting in the specific attachment of lipid‐modified proteins to proteins of interest [50]. This approach has been successfully applied to create target‐specific immunoliposomes, where sortase was used to conjugate antibodies to pentaglycine‐modified liposomes for enhanced cytotoxicity [51]. Microbial transglutaminase (MTG), gaining more prominence in protein lipidation, catalyses irreversible cross‐linking between glutamine and lysine residues in a calcium‐independent manner with broader substrate specificity than mammalian transglutaminase [52, 53, 54]. MTG recognizes specific sequences [55], such as LLQG for glutamine substrates and MRHKGS for lysine substrates, which have been exploited to create water‐soluble lipid‐peptide substrates for efficient MTG‐mediated lipidation under physiological conditions, achieving conversion rates of 80–100% without surfactants [56]. This approach has also shown therapeutic potential *in vivo*, where 40% of cholesterol‐conjugated enhanced green fluorescent protein remained in circulation compared to the native protein [57].

Genetic engineering approaches offer the potential for recombinant production of lipidated proteins, from traditional methods using site‐directed mutagenesis to recent advances in computational design and genetic code expansion that enable direct incorporation of lipidation mimics into virtually any position in the protein sequence, providing unprecedented control over modification sites [58].

### Comparative analysis: choosing the optimal lipidation approach

Successful lipidation designs must balance multiple interdependent factors to achieve the desired therapeutic outcome based on protein and peptide characteristics, administration routes and clinical requirements.

The selection between noncovalent and covalent approaches ultimately depends on the specific therapeutic context (Table 1). Noncovalent HIP offers reversibility, simpler processing and potentially streamlined regulatory pathways. Covalent lipidation provides grated stability in physiological environment, precise control over modification sites and typically longer‐lasting effects.

**Table 1:** Comparative evaluation of the characteristics between covalent and noncovalent lipidation strategies.

Characteristic	Noncovalent lipidation	Covalent lipidation
Bond type	Electrostatic and hydrophobic interactions	Stable chemical bonds, formed by chemical or enzymatic reactions
Stability	Reversible, environment‐dependent	Generally irreversible, stable in physiological conditions
Specificity	Limited site‐specific control	High site‐specificity possible
Release kinetics	Responsive to environmental challenges (pH, ionic strength)	Predetermined by design, less responsive to environment
Complexity	Typically, simpler process	More complex synthesis and purification
Regulatory Considerations	May follow simpler approval pathway	Requires full new drug entity
Manufacturing scale‐up	Often easier to scale	May present complex scale‐up challenges
Cost	Lower overall costs, due to simpler reagents and processes	More expensive due to use of specialized lipid reagents or enzymes
Applications	Primarily used for improved encapsulation and formulation	Widely used for half‐life extension and targeting

The number and position of lipid modifications significantly impact performance [59]. Multiple lipidation sites can enhance membrane interactions and albumin binding but may compromise protein folding and activity. For covalent approaches, spacer/linker design affects both stability and function, with longer linkers potentially improving albumin binding while rigid linkers better maintain protein conformation [60]. Recent research has also demonstrated that peptide dimerization strategies, particularly Y‐shaped configurations, can work synergistically with lipidation to further enhance both potency and plasma stability, offering another dimension for optimizing therapeutic performance [61].

Circular peptides are another promising area for lipidation, combining the enhanced stability of cyclization with the pharmacokinetic benefits of lipid modification [62]. Cyclic peptides extend the druggable target space due to their size, flexibility and hydrogen‐bonding capacity, while offering improved passive membrane permeability compared to their linear counterparts. The strategic combination of cyclization and lipidation addresses multiple therapeutic challenges simultaneously: The cyclic structure provides resistance to exopeptidases and enhanced conformational stability, while lipid modification leads to extended circulation times through albumin binding. One example is murepavadin, a cyclic lipidated peptide that targets *Pseudomonas aeruginosa* infections while simultaneously activating immune cells [63], illustrating the dual functionality possible with cyclic lipidated designs. Similarly, connexin 43‐derived cyclic lipidated peptides achieve half‐lives exceeding 24 h, submicromolar potency and targeted cardiac endothelium delivery [64].

Manufacturing and economic factors also influence lipidation strategy, with noncovalent approaches typically offering cost advantages through simpler reagents and processes compared to the specialized reagents and potentially complex purification required for covalent modifications. Both strategies have proven successful in addressing different delivery challenges, with the optimal choice depending on the target tissue, desired pharmacokinetic profile and manufacturing considerations.

## From structure to function: how lipidation transforms therapeutic properties

**Fig. 3:**
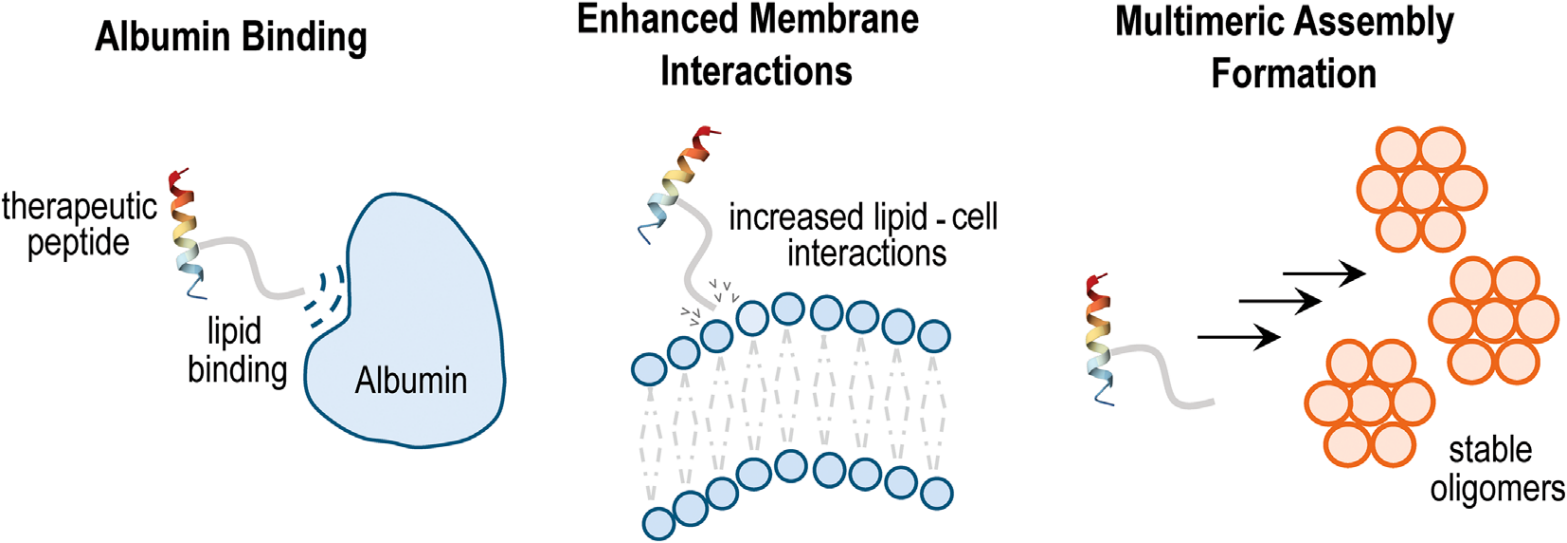
Core mechanisms of action of lipidated peptide and protein therapeutics. Left panel: albumin binding, where the lipid moiety interacts with specific binding pockets in serum albumin, creating a reservoir effect that extends circulation time from hours to days. Centre panel: enhanced membrane interactions, where the amphipathic nature of lipidated peptides facilitates improved cellular uptake and potential crossing of specialized barriers. Right panel: multimeric assembly formation, where lipidated peptides self‐associate into stable oligomeric structures at the injection site, creating a depot effect.

Artificial lipidation fundamentally transforms the behaviour of peptides and proteins in biological systems through distinct mechanistic pathways that address the core limitations of biotherapeutics. By strategically modifying the physicochemical properties of these biologics, lipidation enables extended circulation times, enhanced metabolic stability and improved membrane interactions. These benefits arise through three primary, often complementary mechanisms: albumin binding, enhanced membrane interactions and multimeric assembly formation (Fig. 3).

### Integrated mechanisms of action: a synergistic framework

Human serum albumin (HSA) serves as nature's own drug delivery vehicle and plays a central role in the pharmacokinetics of lipidated therapeutics [65]. As the most abundant circulating protein in plasma, constituting more than half of the total plasma proteins (3.5–5 g·dL^−1^), albumin provides an astonishing circulatory half‐life of 16–18 h and an extended total half‐life of 12–19 days in healthy humans. The interaction between lipidated peptides or proteins and albumin is dominated by hydrophobic interactions between fatty acid chains and specific albumin binding pockets, complemented by electrostatic interactions between carboxylate groups on fatty acids and positively charged albumin residues [66]. To highlight the strength of this interaction, at clinically relevant concentrations, over 99% of liraglutide was found to bind to HSA, which creates a reservoir effect that significantly extends therapeutic duration [67].

The remarkable half‐life extension observed with lipidated therapeutics stems from albumin's ability to evade rapid clearance through its interaction with the neonatal fragment crystallizable receptors (FcRn) [68]. When albumin‐bound drugs enter cells through endocytosis, the FcRn receptor diverts albumin from the lysosomal degradation pathway in a pH‐dependent manner, enabling intact recycling back to circulation. This protective mechanism combined with reduced renal clearance due to the increased size of the albumin‐drug complex dramatically extends the therapeutic window of lipidated conjugates.

Lipidation also significantly enhances cellular uptake and membrane permeability of therapeutic proteins and peptides through increased lipophilicity. The addition of fatty acid moieties creates amphipathic molecules with greater affinity for cellular membranes, enabling several advantageous effects for drug delivery that complement the circulation benefits of albumin binding [69]. The relationship between chain length and membrane permeability follows a complex pattern, suggesting an optimal chain length exists for each specific application and target tissue. A significant advantage of lipidation is the ability to cross specialized barriers, like the blood–brain barrier (BBB), opening opportunities for addressing central nervous system disorders [70].

Complementing these effects, lipidated peptides can form self‐assembling multimeric structures upon subcutaneous injection, creating a local depot effect that further prolongs absorption into the systemic circulation. This has been particularly well‐characterized for insulin analogues such as insulin detemir, where lipidation promotes the formation of dihexamers that dissociate slowly from the injection site [71]. After entering circulation, these molecules then benefit from albumin binding, creating a two‐phase extended‐release profile: initial slow dissociation from the injection site depot, followed by prolonged circulation through albumin binding.

For weekly GPL‐1 receptor agonists like semaglutide, this combined effect of multimeric assembly and albumin binding enables a half‐life extension to approximately 165 h (compared to 1.5 h for native GLP‐1), facilitating once‐weekly dosing regimens that represent significant advances in patient convenience [40]. Similarly, emerging therapies like somapacitan (growth hormone) and insulin icodec leverage these mechanisms to achieve weekly administration schedules that were previously unattainable with standard protein formulations [72, 73, 74].

### Therapeutic implications: translating mechanisms to clinical benefits

The interconnected mechanisms of albumin binding, enhanced membrane interactions and multimeric assembly, work synergistically to overcome the fundamental limitations of protein and peptide therapeutics. The relative contribution of each mechanism varies depending on the specific lipidation strategy, administration route and therapeutic target. For subcutaneously administered therapeutics, the sequential effects of depot formation, followed by albumin binding upon entering circulation, create particularly favourable pharmacokinetic profiles. For orally administered lipidated peptides, the enhanced membrane permeability effects predominate, enabling absorption across intestinal epithelia.

The therapeutic benefits of these mechanisms extend beyond improved pharmacokinetics. By reducing dosing frequency, lipidation enhances patient compliance and convenience. The more consistent plasma levels achieved through these mechanisms can reduce side effects associated with high peak concentrations while maintaining efficacy throughout the dosing interval. Additionally, the potential for tissue‐specific distribution enabled by certain lipidation strategies opens opportunities for targeted drug delivery approaches.

## Therapeutic applications and implications

The mechanistic advantages conferred by artificial lipidation have translated into noteworthy clinical successes across multiple therapeutic areas. By strategically addressing the fundamental limitations of protein and peptide therapeutics, lipidation has enabled significant advances in treatment options, dosing regimens and patient outcomes. This section examines how the physicochemical modifications and mechanistic improvements discussed previously manifest in clinical applications, with particular focus on three key therapeutic benefits: improved pharmacokinetics, enhanced tissue distribution and alternative administration routes.

### Transforming pharmacokinetics: the case of diabetes management

One of the most significant challenges for protein and peptide therapeutics is their rapid clearance from circulation. Most protein and peptide drugs require subcutaneous or intravenous administration as they cross epithelia poorly and are rapidly metabolized. Without half‐life extension strategies, these biologics are typically cleared from serum within minutes to hours through intracellular and extracellular host metabolism and renal filtration.

**Fig. 4:**
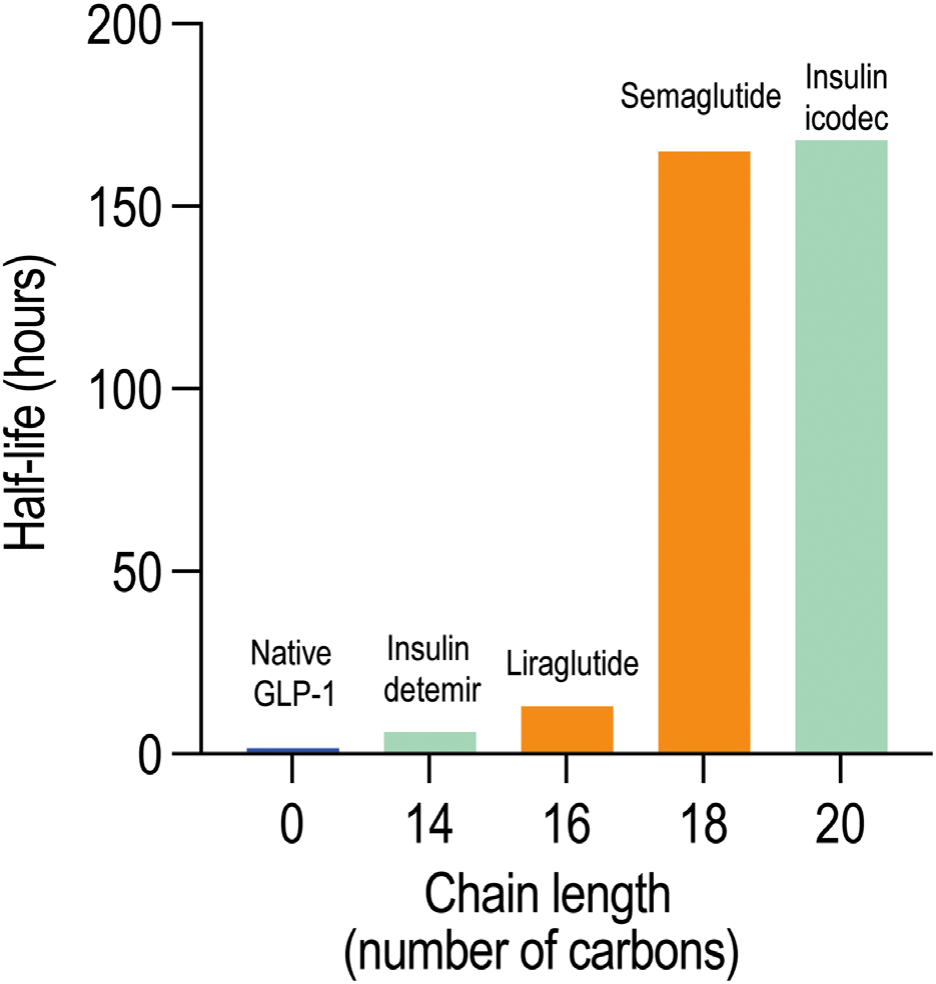
The relationship between lipid chain length and therapeutic half‐life. Native GLP‐1 shows a minimal half‐life while strategic lipidation with progressively longer lipid chains results in exponentially extended circulation times. A significant increase occurs between C16 and C18, enabling the transition from daily to weekly dosing regimens. Orange bars represent GLP‐1 receptor agonists, while green bars indicate insulin analogues.

Lipidation addresses these challenges through the mechanisms detailed in the previous section, with albumin binding being the most significant contributor to extended half‐life. This allows lipidated therapeutics to leverage albumin's intrinsic circulation advantages, as demonstrated by several clinically successful lipidated therapeutics (Fig. 4).

GLP‐1, a hormone with a key role in blood glucose regulation and metabolism, has a short half‐life of approximately 1.5 h due to rapid degradation by dipeptidyl peptidase‐4 (DPP‐4), limiting its therapeutic utility [75]. The progression of GLP‐1 therapy illustrates the transformative impact of lipidation strategies [76]. Exenatide, the first FDA‐approved GLP‐1 receptor agonist derived from Gila monster saliva, required twice‐daily injections due to its relatively short half‐life [77]. Further rational lipidation design strategies have led to unprecedented improvements in patient convenience and therapeutic outcomes as seen with semaglutide that can be administered weekly.

Similar principles have driven advances in insulin therapy. Insulin detemir (Levemir^®^), approved in 2005, was the first lipidated biotherapeutic to reach the market. It consists of desB30 human insulin (lacking threonine at position B30) with C14 myristic acid attached at LysB29. After subcutaneous injection, lipidation promotes self‐association into dihexamers, contributing to slower absorption and increased distribution to peripheral target tissues [78]. Additionally, over 98% of insulin detemir binds to albumin in circulation, extending its half‐life to 5–7 h, compared to regular insulin.

More recently, insulin icodec has been developed with a C20 fatty diacid modification that imparts strong albumin binding, achieving a half‐life of approximately 7 days [73]. This extended duration is further enhanced by three amino acid substitutions (A14E, B16H and B25H) that provide molecular stability and reduce receptor‐mediated clearance [74]. Insulin icodec is administered once weekly, representing a significant advance in insulin therapy, and is approved in the EU under the brand name Awiqli^®^ for both type 1 and 2 diabetes.

### Expanding therapeutic horizons: tissue targeting and alternative routes

Beyond extending circulation time, lipidation can enhance tissue‐specific distribution, and enable alternative administration routes, significantly expanding the therapeutic potential of protein and peptide drugs. A summary of the lipidated therapeutics discussed in this review and their key therapeutic benefits can be seen in Table 2.

**Table 2:** Summary of lipidated therapeutic peptides and proteins and their key features for clinical applications.

Therapeutic (drug)	Mw (kDa)	Lipidation strategy	Key therapeutic benefit	Dosing frequency	Target/application
Insulin detemir	5.9	C14 at LysB29	Half‐life (5–7 h)	Daily	Diabetes
Insulin icodec	6.1	C20 diacid at LysB29	Half‐life (~ 168 h)	Weekly	Diabetes
Liraglutide (GLP‐1)	3.8	C16 at Lys26 via Glu spacer	Half‐life (11–15 h)	Daily	Diabetes (Type 2)
Semaglutide (GLP‐1)	4.1	C18 diacid at Lys26 via ADO spacer	Half‐life (~ 165 h)	Weekly	Diabetes (Type 2)
Somapacitan (Growth hormone)	22.1	Fatty acid via L101C substitution	Half‐life (~ 34 h)	Weekly	Growth hormone deficiency
Lipidated anti‐Tat antibodies	~ 50	Various fatty acids at multiple sites	Enhanced cellular uptake	–	HIV‐1 viral replication
Lipidated octreotide	1.0	C16	Enhanced liver targeting	–	Liver cancer, hepatocellular carcinoma
Lipidated calcitonin	3.4	C16 at cysteine	Enhanced oral bioavailability	–	Oral peptide delivery (proof of concept)
Lipidated leptin peptide [D‐Leu‐4‐OB3]	~ 0.5	C14	86‐fold half‐life improvement	–	Metabolic disorders
Murepavadin (POL7080)	~ 1.7	Cyclic 14‐aa HDP mimetic	Enhanced membrane targeting	–	*P. aeruginosa*

Human growth hormone (hGH) therapy illustrates how lipidation can transform tissue distribution profiles and dosing regimens. Conventional hGH therapy required daily subcutaneous injections due to its short half‐life. Somapacitan (Sogroya^®^), approved by the FDA in 2020 for growth hormone deficiency (GHD), incorporates a single amino acid substitution (L101C) with an attached fatty acid moiety that facilitates reversible albumin binding [72]. This modification extends its *in vivo* half‐life to approximately 34 h in paediatric GHD patients, enabling once‐weekly administration [79].

For liver targeting, lipidated octreotide displays optimized biodistribution with persistent high liver retention, suggesting improved therapeutic potential for liver cancer treatments, particularly hepatocellular carcinoma and liver metastasis [80]. Similarly, modification of human pancreatic polypeptide with palmitic acid results in predominantly liver‐directed biodistribution [81]. This tissue tropism occurs because lipidated peptides bind to serum and liver tissue proteins, while their unmodified counterparts are rapidly cleared via the kidneys.

Perhaps most remarkably, lipidation strategies have been applied to antibody fragments to enhance their ability to reach previously inaccessible tissues [70, 82] Fatty acid lipidated Fab fragments demonstrate the ability to penetrate the BBB and accumulate in brain tissues, likely through transcytosis mechanisms, expanding the scope of therapeutic options for central nervous system disorders with protein therapeutics that would otherwise be excluded from the brain compartment [70]. Furthermore, lipidated antibodies can achieve intracellular delivery, allowing them to bind and functionally inhibit intracellular targets. For example, lipidated anti‐Tat antibodies have been shown to enter living cells and block HIV‐1 replication, demonstrating potential applications in viral diseases [82].

Lipidation also substantially improves membrane permeability, facilitating investigation of noninvasive administration routes for biologics [83]. Several studies have demonstrated successful oral delivery of lipidated peptides, with examples like lipidated salmon calcitonin achieving enhanced oral bioavailability through both increased membrane permeability and proteolytic protection in the gastrointestinal tract [41, 84] and lipidation of leptin‐derived peptide [D‐Leu‐4]‐OB3 with myristic acid leading to an 86‐fold improvement in half‐life extension, dramatically enhancing its therapeutic profile for oral delivery [85].

The relationship between chain length and membrane permeability follows a nonlinear pattern, with optimal chain lengths depending on the specific application [86, 87]. For intestinal absorption models using Caco‐2 cells, short and medium chains typically enhance translocation, while very long chains may impede movement across epithelial barriers [88]. This effect has been exploited to improve oral delivery of biologics, with increases in Caco‐2 monolayer permeability of up to 140‐fold reported for palmitic acid‐derivatized protein conjugates [89]. Some lipidated peptides can even function as their own permeation enhancers through detergent‐like solubilization effects on cell membranes, eliminating the need for separate penetration‐enhancing excipients in formulations [84]. This property makes lipidation particularly valuable for enabling alternative administration routes beyond injection.

While significant challenges remain in achieving reliable and sufficient bioavailability for oral biotherapeutics, lipidation represents one of the most promising approaches for addressing this long‐standing challenge. Continued advances in lipidation strategies, combined with innovative formulation approaches, may eventually enable routine oral administration of protein and peptide therapeutics.

### Clinical considerations and future applications

Further to established clinical applications, lipidation continues to drive innovation in preclinical research. Lipidated antimicrobial peptides are being developed to address resistance [90, 91], while lipidation of urate oxidase shows promise for the treatment of tumour lysis syndrome [92]. In oncology, lipidated peptides show potential for enhanced tissue penetration and prolonged local activity [93]. Neurodegenerative disorders represent another promising application area, with thiopalmitoylated peptides showing enhanced protective effects in multiple sclerosis models through improved bioavailability and amplified anti‐inflammatory responses [94].

Emerging combination approaches that integrate lipidation with other half‐life extension technologies, such as PEGylation or fusion to Fc domains, present opportunities to further enhance therapeutic properties through complementary mechanisms [95, 96]. Similarly, the incorporation of lipidated therapeutics into advanced drug delivery systems, including nanoparticles, liposomes and controlled‐release formulations, can provide additional benefits in terms of stability, targeting and controlled release [22]. The versatility of lipidation approaches, combined with their clinically demonstrated benefits, suggests that we will continue to see expansion of this technology platform across multiple therapeutic areas, with the potential for addressing previously untreatable conditions or improving therapeutic outcomes for existing treatments.

## Challenges and future perspectives

While artificial lipidation has demonstrated success in enhancing the therapeutic potential of proteins and peptides, significant challenges remain in development and implementation. This section examines current technical barriers and emerging solutions, along with future directions that may shape the trajectory of this field.

### Technical and regulatory hurdles in lipidated therapeutics

The translation of lipidated therapeutics from laboratory to commercial scale presents several manufacturing hurdles. For covalent approaches, achieving consistent site‐specific modification remains difficult at industrial scale, particularly for proteins with multiple potential conjugation sites. Heterogeneity in conjugation products can lead to batch‐to‐batch variability, affecting both efficacy and safety profiles. Additionally, removal of unreacted lipids and purification of the final lipidated product requires sophisticated separation techniques that can be challenging to scale. For noncovalent lipidation, maintaining complex stability during formulation processes requires tight control of parameters such as pH, ionic strength and temperature.

Lipidated proteins may exhibit altered conformational stability compared to their native counterparts, presenting formulation challenges [97]. The amphiphilic nature of lipidated conjugates can promote aggregation during storage, potentially compromising long‐term stability and increasing immunogenicity risk. This is particularly problematic for highly concentrated formulations often required for subcutaneous administration. Temperature sensitivity may also be enhanced in lipidated proteins, necessitating cold chain storage and distribution, which increases costs and complicates global access, particularly in resource‐limited settings.

Immunogenicity and regulatory factors are important considerations for clinical development of lipidated therapeutics [98]. Lipidation has been found to modulate the immunogenicity of biotherapeutics, though the effect depends significantly on the specific lipidation approach used [99]. A critical factor is the stability of the bond between the lipid and peptide. Research has found that labile bonds like thioester linkage may enhance immunogenicity *in vivo* compared to more stable amide bonds, suggesting that more stable covalent linkages may be preferable when designing lipidated therapeutics with reduced immunogenicity [99].

The successful development of multiple FDA‐approved lipidated therapeutics demonstrates that immunogenicity concerns can be effectively managed through careful design and optimization of the lipidation strategy. However, predicting immunogenic responses remains challenging due to incomplete understanding of how lipidation affects antigen processing, MHC presentation, and T‐cell activation, requiring extensive preclinical screening and careful clinical monitoring [100].

From a regulatory perspective, covalently modified proteins are classified as new chemical entities, requiring comprehensive safety and efficacy evaluation even when derived from well‐characterized parent proteins [101, 102]. The complexity of these therapeutics often requires the development of multiple orthogonal analytical methods to satisfy regulatory requirements for product characterization. For proteins with multiple potential modification sites, characterizing the distribution of lipidation isomers is particularly challenging but essential for ensuring batch consistency. For noncovalent complexes, determining the precise stoichiometry and orientation of lipid–protein interactions requires advanced biophysical techniques that can be difficult to implement in routine quality control settings and remain an ongoing challenge. Additionally, specific studies to assess the impact of lipidation on pharmacokinetics, biodistribution and potential for drug–drug interactions, particularly for novel lipid modifications or attachment sites may be required.

Economic considerations also significantly impact lipidation technology selection. Noncovalent approaches using HIP typically offer lower costs through inexpensive surfactants and a simplified process, while covalent lipidation generally requires more expensive specialized reagents and potentially costly enzymatic processes that may demand frequent replacement due to sensitivity and degradation.

### Emerging solutions and future directions

Recent advances in biorthogonal chemistry are addressing the site‐specificity challenges [103]. Enzymatic approaches using engineered transglutaminases and sortases enable highly specific conjugation under mild conditions, reducing heterogeneity in the final product. These enzymatic methods offer advantages in terms of reaction specificity and efficiency, potentially simplifying downstream purification. Genetic engineering approaches, including incorporation of noncanonical amino acids, allow even more precise control over modification site and stoichiometry by introducing biorthogonal handles at predetermined positions [58, 104]. Computational modelling and simulation techniques can predict optimal modification sites that maximize the therapeutic benefits while minimizing stability or activity impacts and potentially reduce development timelines and costs [105, 106].

Novel lipid structures with tailored properties represent another frontier in lipidation technology. Branched and dendritic lipids offer unique physicochemical properties that can enhance albumin binding [107]. Stimuli‐responsive lipid conjugates that release the therapeutic protein under specific physiological conditions (pH, enzymatic activity, redox environment) could enable tissue‐specific drug release, potentially enhancing therapeutic index and reducing off‐target effects [108].

Nanotechnology‐based approaches are increasingly combined with lipidation to address formulation challenges. Hydrophobic ion pairing has enabled successful encapsulation of hydrophilic peptides into lipid‐based nanocarriers [22, 26, 27, 28]. Diverse applications demonstrate the versatility of this technique, including antimicrobial peptide delivery via melittin–sodium dodecyl sulphate complexes in PLGA nanoparticles [109], targeted cancer therapy using lycobetaine–oleic acid nanoemulsions with enhanced antitumour activity [110] and improved chemotherapy delivery through a vincristine‐oleic acid submicron emulsion [111]. Covalent lipidation in polymer–lipid nanoparticles achieved up to 35‐fold improved encapsulation through palmitic acid conjugation of peptide antigens and adjuvants [112]. Recent innovations include adaptor lipids for mRNA‐loaded lipid nanoparticles [95], charge‐converting nanostructured carriers containing a lipidated cell‐penetrating peptide for enhanced cellular uptake [113] and palmitoylated peptides enabling efficient siRNA delivery through cationic lipid nanocarriers with optimized lamellar structures [114], demonstrating the expanding scope of lipidation in advanced nucleic acid delivery systems.

Advanced analytical technologies have improved characterization capabilities for lipidated therapeutics. Native mass spectrometry has emerged as a powerful tool for analysing lipidated proteins in their natural conformations, allowing researchers to study binding interactions, stoichiometry and structural integrity [115].

The clinical success of existing lipidated therapeutics has established artificial lipidation as a validated technology platform with significant commercial impact. The GLP‐1 receptor agonist market worth alone is projected to exceed $55 billion by 2031, driven primarily by lipidated analogues like semaglutide [116]. The pharmaceutical industry's continued investment in lipidation technologies is evident from the robust and growing pipeline of lipidated candidates across multiple therapeutic areas.

Looking forward, the most promising directions include orally bioavailable lipidated proteins, multi‐functional lipidated constructs for targeted delivery and precision medicine applications tailored to individual patient needs. Patient‐specific optimization of lipidation properties based on individual pharmacokinetic profiles could enable truly personalized dosing regimens. Additionally, the combination of lipidation with advanced drug delivery technologies promises to further enhance therapeutic outcomes.

## Conclusion

This review has traced the journey of artificial lipidation from fundamental chemical strategies through mechanistic understanding to clinical applications and finally to current challenges and emerging solutions. Artificial lipidation has transformed protein and peptide therapeutics, enabling unprecedented improvements in pharmacokinetics, tissue distribution and administration routes. The extraordinary clinical success of lipidated GLP‐1 agonists, insulins and growth hormone analogues underscores the therapeutic potential of this technology platform.

Despite the technical and regulatory challenges, the field of artificial lipidation continues to advance rapidly, driven by clinical need and commercial opportunity. The fundamental principles established through early successes provide a strong foundation for next‐generation lipidated therapeutics with even more sophisticated properties and expanded therapeutic applications. As researchers continue to elucidate the structure–activity relationships and optimize lipidation strategies, this technology platform will likely remain at the forefront of biotherapeutic innovation, offering new solutions for previously intractable medical challenges.

## Conflict of interest

The authors declare no conflict of interest.

## Author contributions

SC and JM contributed to conceptualization, review and editing; SC, JM and EV contributed to writing – original draft; and SC contributed to supervision.
